# Potential predictive value of CT radiomics features for treatment response in patients with COVID‐19

**DOI:** 10.1111/crj.13604

**Published:** 2023-03-21

**Authors:** Gang Huang, Zhongyi Hui, Jialiang Ren, Ruifang Liu, Yaqiong Cui, Ying Ma, Yalan Han, Zehao Zhao, Suzhen Lv, Xing Zhou, Lijun Chen, Shisan Bao, Lianping Zhao

**Affiliations:** ^1^ Department of Radiology Gansu Provincial Hospital Lanzhou Gansu China; ^2^ The Department of CT Tianshui Combine traditional Chinese and Western Medicine Hospital Tianshui Gansu China; ^3^ GE Healthcare China Beijing China; ^4^ Clinical Medical School Gansu University of Chinese Medicine Lanzhou Gansu China; ^5^ Ward II of Respiratory Medicine The First Hospital of Tianshui Tianshui Gansu China; ^6^ Department of Radiology The First Hospital of Tianshui Tianshui Gansu China; ^7^ School of Medical Sciences The University of Sydney Sydney New South Wales Australia

**Keywords:** COVID‐19, disease prognosis, radiomics, tomography, X‐ray computed

## Abstract

**Introduction:**

This study aims to explore the predictive value of CT radiomics and clinical characteristics for treatment response in COVID‐19 patients.

**Methods:**

Data were collected from clinical/auxiliary examinations and follow‐ups of COVID‐19 patients. Whole lung radiomics feature extraction was performed at baseline chest CT. Radiomics, clinical, and combined features (nomogram) were evaluated for predicting treatment response.

**Results:**

Among 36 COVID‐19 patients, mild, common, severe, and critical disease symptoms were found in 1, 21, 13, and 1 of them, respectively. Twenty‐five (1 mild, 18 common, and 6 severe) patients showed a good response to treatment and 11 poor/fair responses. The clinical classification (*p* = 0.025) and serum creatinine (*p* = 0.010) on admission and small area emphasis (*p* = 0.036) from radiomics analysis significantly differed between the two groups. Predictive models were constructed based on the radiomics, clinical features, and nomogram showing an area under the curve of 0.651, 0.836, and 0.869, respectively. The nomogram achieved good calibration.

**Conclusion:**

This new, non‐invasive, and low‐cost prediction model that combines the radiomics and clinical features is useful for identifying COVID‐19 patients who may not respond well to treatment.

## Graphical abstract

Can radiomic and clinical characteristics method improve the prediction of treatment response in COVID‐19 patients? This study found that small area emphasis and serum creatinine levels have significance in different treatment response groups. It is suggested that the combined model can improve the prognostic prediction and dynamic clinical process analysis finding more potential valuable indicators.

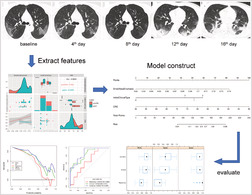

## 1 INTRODUCTION

Since the outbreak of COVID‐19 in Wuhan, China, that occurred in December 2019,^1^ extraordinary measures taken in China have led to a decline in COVID‐19 cases.^2^ However, the virus has spread to many countries across the globe during that time, causing 214 million infections and almost 5 million deaths (data from August 27, 2021) (https://covid19.who.int/). At present, the main therapeutic medications for COVID‐19 include antiviral vaccines, antibiotics, glucocorticoids, convalescent plasma, hyperimmune immunoglobulins, immunomodulatory therapy, and traditional Chinese medicines.^3^ Yet, a shortage of medicines and poor therapeutic effects are currently the main limitations of COVID‐19 therapy. In addition, maintenance therapy with a ventilator is the only available effective approach when glucocorticoids fail. Hence, it is essential to establish a treatment response or prognostic prediction model for individuals affected by COVID‐19.

Most COVID‐19 patients present with mild clinical manifestations, and those who develop severe symptoms usually have a poor prognosis.^4^ A recent study reported that the prevalence of severe COVID‐19 ranges from 15.7% to 26.1%; these patients show abnormal chest computed tomography (CT) findings and clinical laboratory tests.^4^, ^5^, ^6^ Although the diagnostic value of chest CT for COVID‐19 is non‐specific and overlaps with other chest infections, the CT image's detailed information could provide a useful clue for the treatment response or prediction of the disease.

So far, a number of studies have reported on chest CT data in COVID‐19 patients^7^, ^8^, ^9^, ^10^, ^11^; most were based on diagnosis^10^, ^11^ and severity assessment.^7^, ^9^ In addition, most recent studies have focused on examining qualitative^10^, ^12^ and semi‐quantitative scores of CT images.^10^, ^11^ A score from 0 to 5 was given for each lung lobe and was used to evaluate pulmonary involvement. However, quantitative analysis of chest CT provides an objective approach to the clinical evaluation and management of inflammatory or infiltrative lung diseases.^13^

Radiomics is a relatively new quantitative and objective technique that extracts many features from radiographic medical images using data‐characterization algorithms.^14^, ^15^ Data extracted from CT images offer non‐invasive profiling of lesion characteristics, such as tumor heterogeneity.^16^, ^17^ In addition, they can be very useful in assessing pneumonia features,^18^, ^19^ as they could assist in accurate diagnosis, prognosis evaluation, and longitudinal management of the disease.

Recently, several studies have assessed the feasibility of radiomics‐based prognostic prediction.^20^, ^21^ Combined with clinical features, radiomics signatures have been significantly associated with survival rate in non‐small cell lung cancer patients,^22^ suggesting that radiomics signature is an independent biomarker for the estimation of disease‐free survival in patients with early‐stage non‐small cell lung cancer.^15^ Therefore, in this retrospective study, we reviewed valuable radiomics and quantitative index features in confirmed COVID‐19 patients to evaluate the predictive value of early chest CT and clinical risk factors for the progression and prognosis of COVID‐19, which could provide decision support regarding the prognosis and longitudinal evaluation of the disease.

## 2 MATERIALS AND METHODS

### 2.1 Ethical statement

The present study was approved by the Human Ethical Committee of our hospital (Approval number: 2020‐012), and the requirement for informed consent was waived due to the retrospective nature of the study.

### 2.2 Patients

This retrospective study included 37 patients diagnosed with COVID‐19 between January 23, 2020, and March 2, 2020. The inclusion criteria were: (1) COVID‐19 confirmed by PCR (two positive results were required); (2) available chest CT scan within 5 days after admission. Exclusion criteria were the following: (1) no lesions detected by chest CT scan; (2) have fundamental pulmonary disease. Discharge and release quarantine criteria were: (1) temperature returned to normal for more than 3 days; (2) respiratory symptoms were significantly relieved; (3) abnormal infammation CT imaging findings substantially absorbed; (4) the PCR test was negative for two consecutive respiratory pathogens (sampling interval ≥1 day). At the time of writing, 34 patients were recovered, and three remained in the hospital.

### 2.3 Clinical and image data collection

Available clinical history, laboratory, CT scanning data, treatment, and prognosis information were collected. Depending on their clinical conditions, all patients received antiviral (lopinavir and tonavir) therapy, supportive care, a traditional Chinese herb, antibiotics, and corticosteroid. Invasive mechanical ventilation was used for critical cases. Patients were characterized as mild, common, severe, and critical types based on the guideline of COVID‐19 (Trial Version 7).^23^ In addition, responses to clinical treatment were defined as (1) good, symptoms relieved; (2) fair, symptoms are not relieved or relapsed; (3) poor, symptoms aggravated.

### 2.4 Imaging acquisition

Subjects underwent multidetector CT spiral scans with 16 detector rows (Emotion 16; Siemens, Erlangen, Germany; Aquillion™ 16; Toshiba Medical Systems Corp, Tokyo, Japan) or dual‐source CT instrument (Siemens SOMATOM Definition; Flash, Germany) with the following scanning conditions: (1) 120 kVp; automatic milliamperes; pitch, 1.2; and 0.6‐s rotation; (2) 120 kV; 120 mAs; pitch 1.33; and 0.6‐s rotation; (3) 120 kVp; CareDose 4D intelligent mode, pitch, 1.2; and collimation, 128 × 0.6 mm, respectively. All imaging data were reconstructed using a lung sharp reconstruction algorithm with a thickness of 1.25 mm. CT images were acquired in the supine position at full inspiration; the scanning direction was from the head to the foot.

### 2.5 Chest CT data processing and texture analysis

The CT images were analyzed by two experienced radiologists independently. The lung volume was extracted by the Otsu algorithm performed on lung intelligence kit software (LK; version 1.1.0, GE Healthcare, China). Radiomics features were extracted using PyRadiomics (version 2.2.0, https://pyradiomics.readthedocs.io),^24^ which is an open‐source radiomics toolbox. Ninety‐three texture features from the category of first‐order statistical features (histogram), gray‐level co‐occurrence matrix (GLCM) features, gray‐level run‐length matrix (GLRLM) features, gray‐level size zone matrix (GLSZM) features, gray‐level dependence matrix (GLDM) features, and neighboring gray‐tone difference matrix (NGTDM) were extracted from baseline CT image. In addition, the percentage of lung lesions in total lung volume was calculated using segment statistics in 3D Slicer software (version 4.10.2, https://www.slicer.org). A detailed description of the radiomics features, the calculation of the lung lesion volume, and the percentage are shown in the Supporting Information.

### 2.6 Construction of prognostic models

We performed a three‐step procedure to select the most important and robust radiomics features. First, features with a *p*‐value <0.05 in univariable analyses were selected. Next, the Spearman correlation test was performed to remove correlation values >0.8. Finally, multivariable logistic regression analysis was used to determine the association between different radiomics features. The backward stepwise logistic regression was used to select the best variables, and Akaike's information was used as a stopping criterion. Clinical factors, including age, sex, pulmonary lesion volume and percentage, incubation period, temperature, initial oxygen saturation, initial clinical type, complications, and laboratory findings, were first selected through univariable analysis. The important factors, which included initial clinical type and serum creatinine levels, remained in a multivariable logistic regression model. The select radiomics features and important clinical factors were then combined to fit a multivariable logistic regression model. Finally, the multivariable logistic regression model was visualized as the nomogram.

### 2.7 Statistical analysis

All statistical analyses were performed in R software (version 3.6.1, https://www.rproject.org). Categorical variables were expressed as numbers or percentages, and continuous variables were expressed as mean (standard deviation, SD) or median (interquartile range, IQR). The Student *t*‐test was used to determine whether the values of the normally distributed demographic and clinical variables significantly differed between the good and poor/fair group. A Mann–Whitney U test was used for the continuous variable with the abnormal distribution. The receiver operating characteristic curve (ROC) and area under curve (AUC) were used to evaluate the model's classification performance. A Bootstrap approach with resampling 200 times was used to calculate AUC, sensitivity, and specificity. Calibration curves accompanied by the Hosmer–Lemeshow test were performed to assess the models. Decision curve analysis was also used to evaluate the clinical usefulness. A two‐tailed *p*‐value <0.05 indicated statistical significance. The ROC plots were generated using the “pROC” package; the nomogram was plotted using the “rms” package; DCA was performed using the “dca. R” in R software.

## 3 RESULTS

### 3.1 Basal clinical characteristics

A total of 36 patients were included in the study; 1 patient was excluded due to a lack of initial CT scan data. Among 36 COVID‐19 patients, mild, common, severe, and critical disease symptoms were found in 1, 21, 13, and 1 of them, respectively. Twenty‐five (1 mild, 18 common, and 6 severe) patients showed a good response to treatment, 7 (4 common and 3 severe) were fair responses, and 4 (3 severe and 1 critical) were poor responses. There was a significant difference in initial clinical type (*p* = 0.025) between the two groups. After reviewing the disease process, significant differences in the maximum temperature (*p* < 0.001) and hospitalized period (*p* = 0.034) were also found between the two groups (Table 1). Among a total of 22 laboratory tests at baseline, serum creatinine was the only significant difference detected between the good and poor/fair group (Table 2); it was substantially higher in the poor/fair than in the good group (*p* = 0.010) (Tables 1 and 2).

**TABLE 1:** Demographic data and basal clinical characteristics in poor/fair and good treatment response group of COVID‐19 patients.

	Poor/fair group	Good group	*P* values
Demographics and clinical characteristics			
Number	11	25	N/A
Sex (female), N (%)	5(45.5%)	15(60.0%)	0.770^a^
Age, mean (SD)	57.60 (22.33)	48.17 (16.91)	0.187^b^
Incubation period, median (IQR)	9.00 (8.00, 10.00)	10.00 (7.25, 10.00)	0.652^c^
Temperature, mean (SD)	36.54(0.27)	36.77(0.57)	0.404^b^
Initial oxygen saturation, mean (SD)	95.25(2.99)	94.69(3.63)	0.779^b^
Number of fever, N (%)	7(63.6%)	16(64.0%)	0.983^a^
Febrile days, mean (SD)	7.00(3.46)	7.31(6.83)	0.719^b^
Number of cough, N (%)	9(81.8%)	14(56.0%)	0.137^a^
Days of cough, mean (SD)	11.78(6.38)	10.79(6.77)	0.134^b^
Initial clinical type, N (%)			0.025^b^ ^,^ ^*^
Mild	0	1 (4.0%)	
Common	4 (36.4%)	17 (68.0%)	
Severe	6 (54.5%)	7 (28.0%)	
Critical	1 (9.1%)	0	
Hospitalized period, mean (SD)	15.30(5.01)	15.09(4.73)	0.034^b^ ^,^ ^*^
Maximum temperature, mean (SD)	38.77(0.96)	38.29(0.79)	<0.001^b^ ^,^ ^*^
Complication, N (%)	4 (36.4%)	2 (8.7%)	0.134^a^

*Note*: Values are reported as mean standard deviation (SD), median interquartile range (IQR) or numbers and percentages.

Abbreviations: IQR, interquartile range; SD, standard deviation.

^a^ Chi square test.

^b^ Two‐sample *t* test.

^c^ Mann–Whitney test.

* *P*＜0.05.

**TABLE 2:** Basal laboratory findings and pulmonary involvement on chest CT in poor/fair and good treatment response group of COVID‐19 patients.

	Normal range	GoodMean (SD) or median (QRI)	Fair/poorMean (SD) or median (QRI)	*P* values
Laboratory findings				
WBC, ×10^9^/L	4–10	3.94 (2.29)	4.59 (1.24)	0.436
NEU, ×10^9^/L	2.0–7.0	2.43 (2.08)	2.93 (1.00)	0.500
LYM, ×10^9^/L	0.8–4.0	1.15 (0.73)	1.07 (0.32)	0.778
MON, ×10^9^/L	0.12–1	0.32 (0.30, 0.40)	0.50 (0.36, 0.63)	0.063
PLT, ×10^9^/L	85–303	148.50 (64.83)	150.27 (92.74)	0.953
HGB, g/L	113–151	135.65 (16.41)	137.67 (17.94)	0.768
GLU, mmol/L	3.89–6.11	5.45 (1.21)	5.48 (2.07)	0.963
PCT, ng/mL	0.00–0.5	0.17 (0.09)	0.72 (0.89)	0.080
CRP, mg/L	2–8	17.66 (17.04)	26.96 (28.19)	0.314
D‐dimer, μg/L	0.00–1.00	0.43 (0.50)	0.24 (0.19)	0.344
ESR, mm/h	0–15	20.46 (18.97)	14.20 (11.82)	0.505
UREA, mmol/L	2.7–8.3	4.32 (1.88)	5.39 (2.59)	0.255
CRE, μmol/L	31.8–93.7	59.00 (53.50, 68.50)	72.00 (68.75, 88.42)	0.010*
TB, μmol/L	2–20	12.18 (5.30)	13.82 (7.30)	0.528
ALB, g/L	30–55	38.51 (4.79)	42.40 (2.61)	0.099
ALT, U/L	0–42	32.88 (35.91)	28.20 (9.15)	0.779
AST, U/L	0–38	29.76 (10.03)	22.83 (4.62)	0.121
CK, U/L	0–190	87.87 (69.96)	67.67 (31.47)	0.509
LDH,U/L	115–220	209.88 (75.07)	190.00 (42.87)	0.604
APTT, sec	21–38	38.88 (4.01)	38.56 (5.68)	0.891
PT, sec	13–21	11.01 (1.67)	11.08 (0.98)	0.933
Lung lesion volume and percentage from chest CT				
Lesion volume, cm^3^		276.22 (299.03)	299.87 (298.35)	0.828
Lesion percentage, %		0.08 (0.11)	0.09 (0.08)	0.859

*Note*: Values are reported as mean standard deviation (SD), median interquartile range (IQR).

Abbreviations: ALB, albumin; ALT, alanine aminotransferase; APTT, activated partial thromboplastin time; AST, aspartate aminotransferase; CK, creatine kinase; CRE, creatinine; CRP, C‐reactive protein; ESR, erythrocyte sedimentation rate; GLU, glucose; HGB, hemoglobin; IQR, interquartile range; LDH, lactate dehydrogenase; LYM, lymphocyte; MON, monocyte; NEU, neutrophil; PCT, procalcitonin; PLT, platelet; PT, prothrombin time; SD, standard deviation; TB, total bilirubin; UREA, serum urea nitrogen; WBC, white blood cell.

### 3.2 Construction of the radiomics based predictive modeling

**FIGURE 1:**
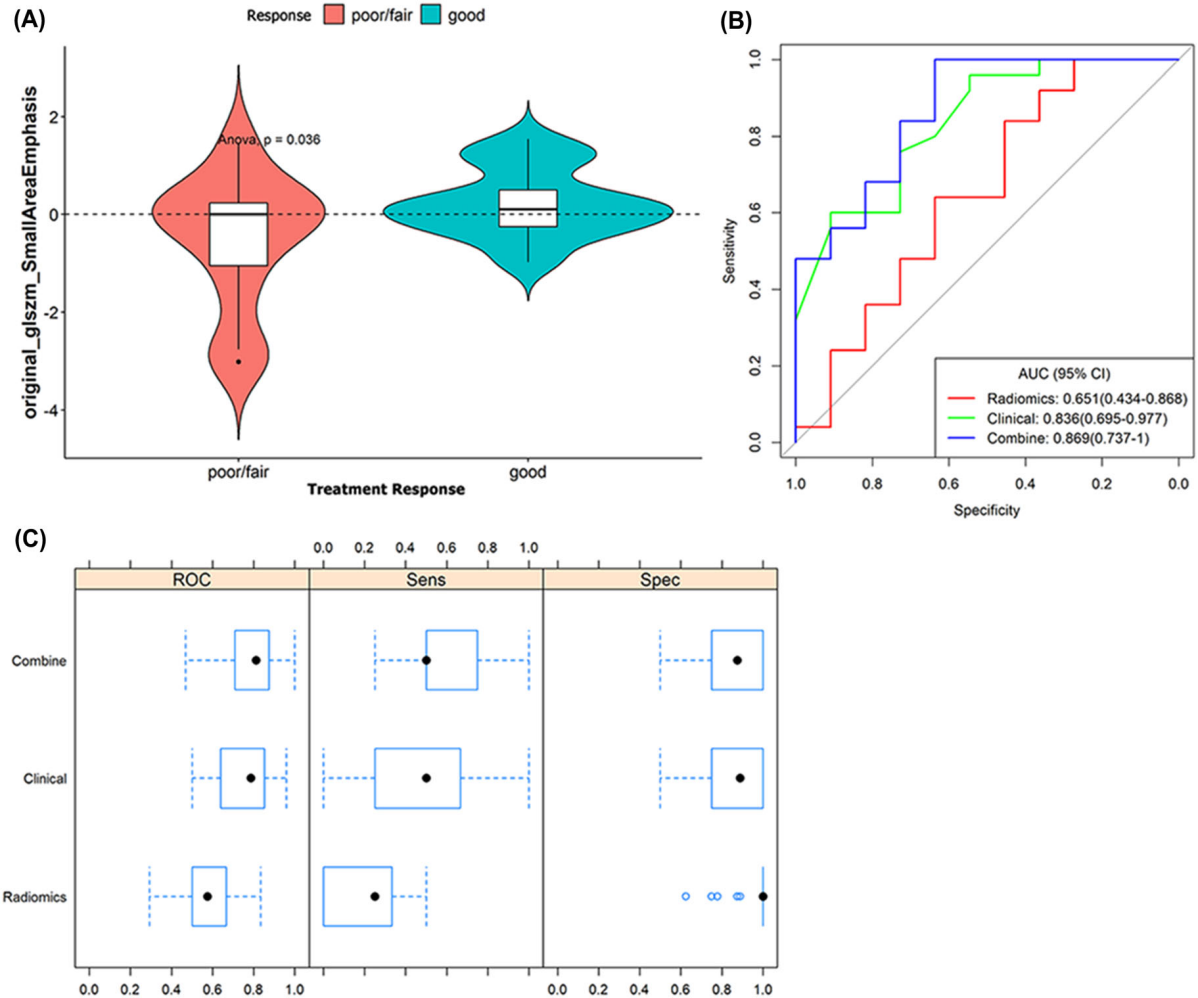
(A) Violin and box plots are showing a lower small area emphasis from the radiomic feature in a poor/fair treatment response group compared with a good treatment response group. (B) The receiver operating characteristic (ROC) curve of the radiomics, clinical, and the combined predictive model for the treatment response of COVID‐19, respectively; the red line represents the area under the curve (AUC) of the radiomics model, the green line represents the AUC of the clinical model, and the blue line indicates the AUC of the combined model. (C) Compare and analysis of the sensitivity and specificity of the three predictive models for the treatment response.

The small area emphasis (*p* = 0.036) in radiomics features (Figure 1A) and the serum creatinine levels (*p* = 0.010) were significant in the poor/fair group. The definition of small area emphasis is shown in Appendix (Supporting Information). A predictive model for the treatment response to COVID‐19 was established using multivariable analysis. Small area emphasis was the best‐selected predictor for the texture analysis, with an AUC of 0.651 and a sensitivity and specificity of 0.84 and 0.455, respectively. Initial clinical type and serum creatinine levels were the best‐selected predictors for clinical characteristics, with an AUC of 0.836 and a sensitivity and specificity of 0.600 and 0.909, respectively. The AUC improved to 0.869 (Figure 1B) after combining all parameters. We found the radiomics model (small area emphasis) had higher sensitivity, whereas the clinical feature model had higher specificity (the best cut‐off value, sensitivity or specificity was 0.648, 84.0%, or 45.5%, respectively, for radiomics model, and 0.847, 60.0% or 90.9%, respectively, for clinical model). Thus these two models had complementary effects when combined (the best cut‐off value, sensitivity, and specificity were 0.368, 100%, and 63.6%, respectively), as shown in Figure 1C. The detailed pair‐wise relationship between the three statistically significant deferent indicators is shown in Figure S1.

**FIGURE 2:**
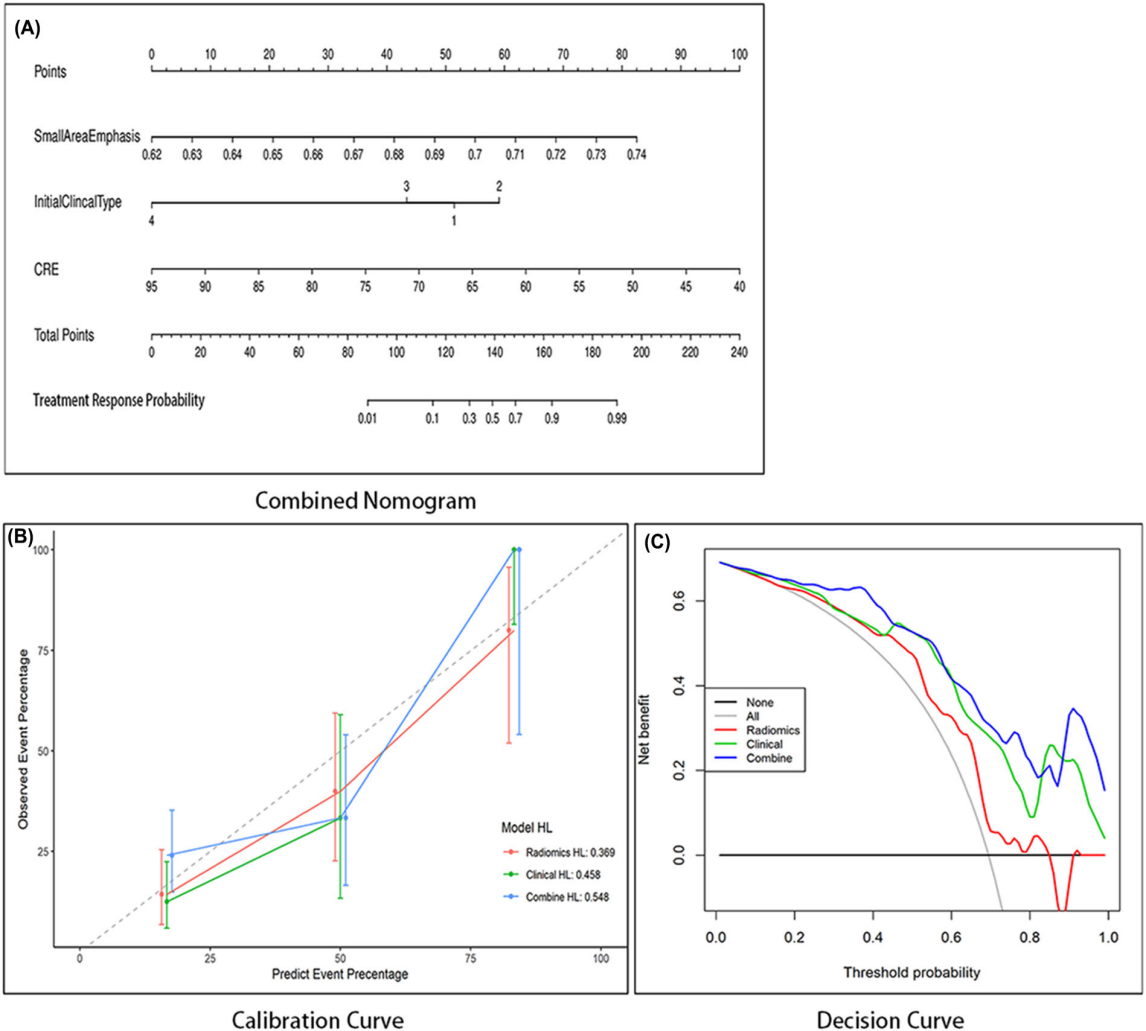
(A) The combined nomogram of radiomics (small area emphasis) and clinical model (initial clinical type and serum creatinine [CRE]) for predicting the treatment response of COVID‐19 patients. The lower total score represents a lower probability of good treatment response. (B) Calibration plot for the three prediction models. The red line represents the radiomics model, the green line represents the clinical model, and the blue line represents the combined model. (C) Decision curve analysis for each model. The y‐axis measures the net benefit, and the x‐axis represents the threshold probability. The combined model had the highest net benefit compared with the other two models.

A nomogram incorporating the significant predictors from the multivariable analysis was then established (Figure 2A). Small area emphasis (Figure S2, supplementary material), initial clinical type, and serum creatinine levels, which were shown to be independent predictive factors in the multivariable logistic regression analysis, were included in the nomogram. By summing the scores of each variable, we predicted the different treatment responses. The calibration curve showed that the nomogram was well‐calibrated, and the Hosmer–Lemeshow test yielded a non‐significant *p* of 0.369, 0.458, and 0.548, describing the good fit of the model (Figure 2B).

The decision curve analysis showed that the combined nomogram had a higher overall net benefit than the radiomics or the clinical model at the threshold probability of 21%–46%, which indicates that within this range, the combined nomogram outperformed the radiomics or clinical features with more accuracy in response prediction. The results indicate that the combined nomogram is a reliable clinical treatment tool to predict treatment response in patients with COVID‐19 (Figure 2C).

### 3.3 Dynamic clinical process of good and poor/fair response group

**FIGURE 3:**
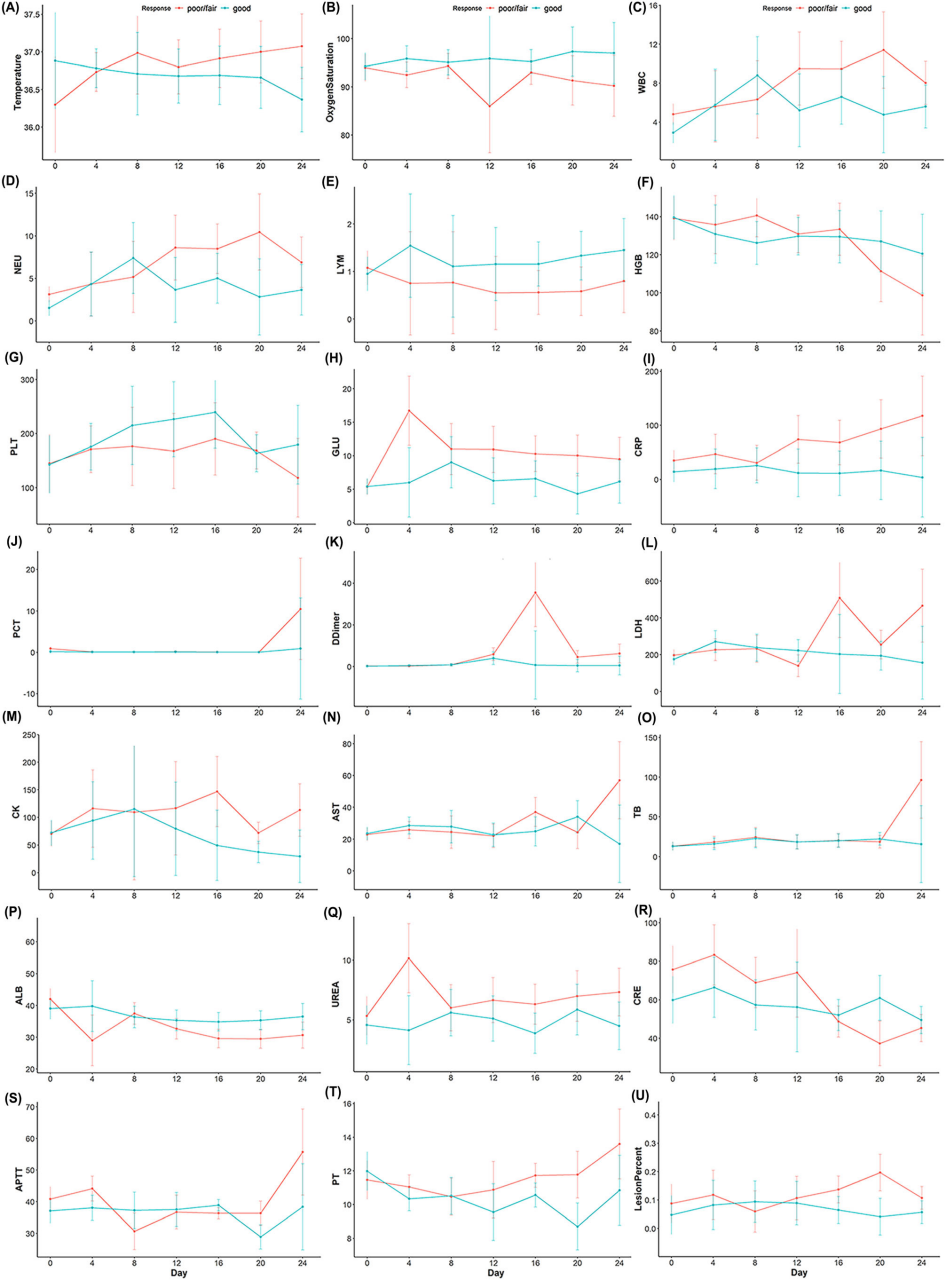
The dynamic curve of clinical variables, laboratory findings (including hematological and biochemical parameters), and pulmonary lesion percentage on chest CT in the disease course of the poor/fair and good treatment response group in COVID‐19. (A, B) temperature and oxygen saturation. (C–H) routine blood test parameters. (I–K) Inflammatory response‐related indicators. (L–M) cardiac function‐related indicators. (N–P) Liver function‐related indicators. (Q–R) Renal function‐related indicators. (S–T) Coagulation function‐related indicators. (U) Pulmonary lesion percentage on chest CT of the two groups. ALB, albumin; APTT, activated partial thromboplastin time; AST, aspartate aminotransferase; CK, creatine kinase; CRE, creatinine; CRP, C‐reactive protein; GLU, glucose; HGB, hemoglobin; LDH, lactate dehydrogenase; LYM, lymphocyte; NEU, neutrophil; PCT, procalcitonin; PLT, platelet; PT, prothrombin time; TB, total bilirubin; UREA, serum urea nitrogen; WBC, white blood cell.

To determine the clinical characteristics during COVID‐19 progression, the dynamic changes of clinical variables (including temperature and oxygen saturation), clinical laboratory parameters (including hematological and biochemical parameters), and pulmonary lesion volume and percentage on chest CT were tracked from day 1 to 24 after the onset of the disease at average 4‐day intervals. At the end of March 5, 2020, data from 36 patients with the complete clinical course were analyzed (Figure 3). During hospitalization, the oxygen saturation was lower, but the temperature was higher after the 8th day of admission in the poor/fair group compared with the good group. In terms of laboratory tests, most patients had marked lymphopenia, particularly in the poor/fair group (all *P* < 0.05). White blood cell counts, neutrophil counts, and serum glucose were higher, but hemoglobin and platelets were lower in the poor/fair group than those in the good group (all *P* < 0.05). D‐dimer and C‐reactive protein levels were higher in the poor/fair group than those in the good group, and the procalcitonin was significantly elevated on the 20th day of hospitalization in the poor/fair group (all *P* < 0.05).

**FIGURE 4:**
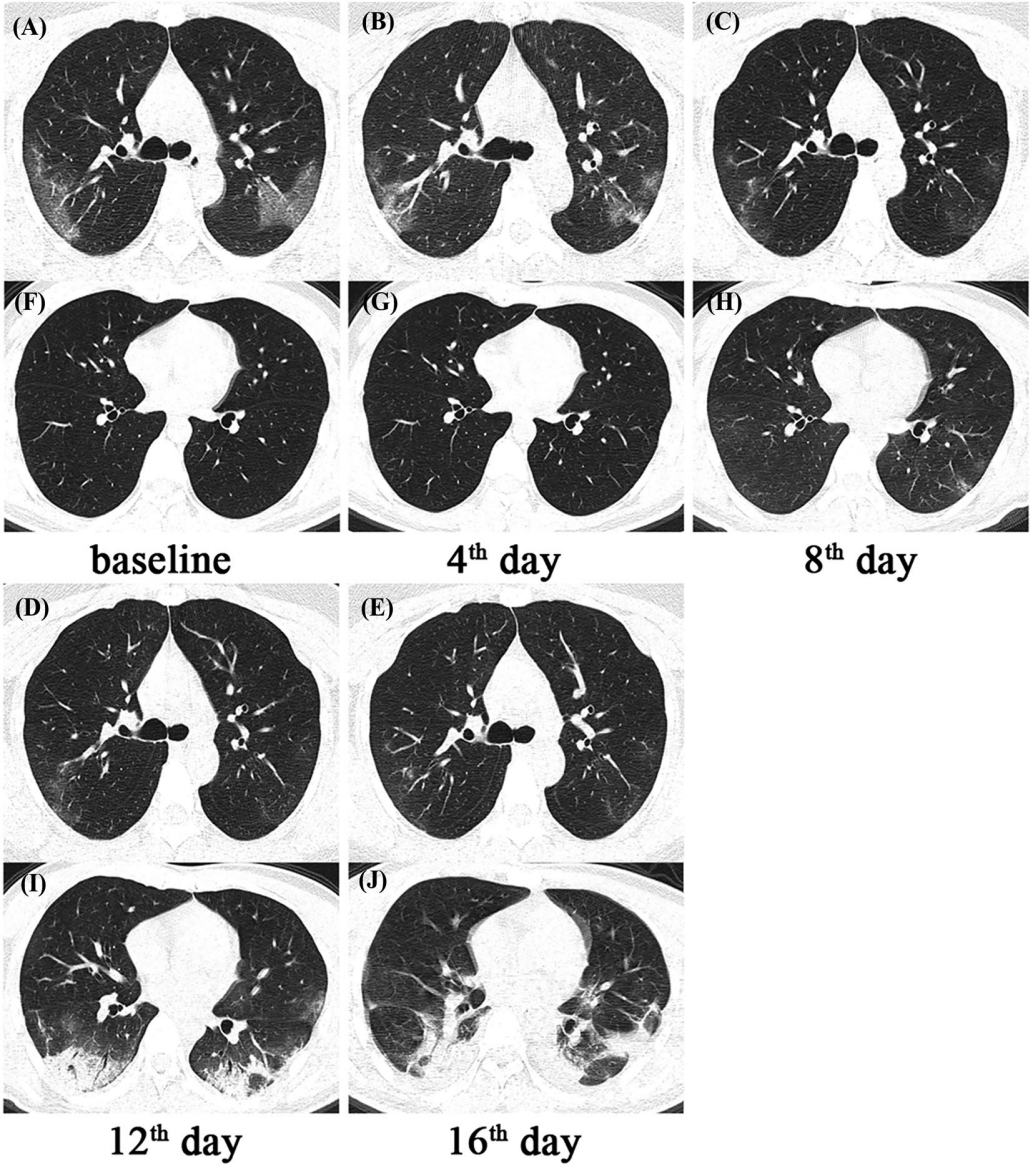
On chest CT, there is a dynamic change in the pulmonary lesions. (A–E) A 33‐year‐old male COVID‐19 patient responded well to treatment; his lung lesions were gradually absorbed. (F–J) A 34‐year‐old male COVID‐19 patient with a poor treatment response; his lung lesion on the CT 4 days after admission was almost invisible to the naked eye. However, the lesions were significantly advanced (H–I) with bilateral pleural effusion (J) in the later stage, particularly in the lungs' peripheral area.

Similarly, as the disease progressed and clinical status deteriorated, the indicators reflecting heart (creatine kinase and lactate dehydrogenase), liver (aspartate and alanine aminotransferase, total bilirubin), kidney (urea and creatinine), and coagulation (activated partial thromboplastin time and prothrombin time) increased, and the albumin decreased in poor/fair group. Not surprisingly, the lesion volume and percentage of chest CT were larger in the poor/fair group than those in the good group (Figure 4).

## 4 DISCUSSION

In this study, we constructed three prognostic models for predicting the treatment response in COVID‐19 patients based on the radiomics and clinical features. The nomogram (combined model) showed the highest AUC compared with the other two and yielded the best predictive value for the treatment response.

Treatment response evaluation is essential in treatment decision‐making. Huang et al.^15^ reported that the radiomics signature is an independent biomarker for estimating disease‐free survival in patients with early‐stage non‐small cell lung cancer. Moreover, Bak et al.^25^ used texture‐based quantitative CT features for clusters and assessed the predicted clinical outcome in patients with idiopathic pulmonary fibrosis. Aerts et al.^26^ reported that the CT radiomic features before treatment could predict epidermal‐growth factor‐receptor mutation status in non‐small cell lung cancer and are associated with gefitinib response. Our data is consistent with previous findings, suggesting an important role of radiomics in predicting pulmonary disease. In the present study, we applied radiomics to the prediction of treatment response to COVID‐19. A texture feature named small area emphasis (Figure S2), which belongs to GLSZM for measuring the distribution of small size zones (a greater value indicative of smaller size zones and more fine textures^27^), was significantly decreased in the poor/fair treatment response group, with good sensitivity (84.0%), relatively low specificity (45.5%) and a cut‐off of 0.648.

In the present study, the clinical features selected in the predictive model as predictors were serum creatinine levels and clinical type on admission. The creatinine level of all patients in this cohort did not exceed the normal range, but it was higher in the poor/fair treatment response group than in the good treatment response group, which is consistent with previous studies^4^, ^5^ demonstrating that the elevated creatinine is associated with the death of COVID‐19. Interestingly, in the dynamic clinical observation of serum creatinine, the average creatinine level decreased in the poor/fair group after 15 days of admission, which might be related to the active clinical interventions. Considering that 76% (19/25) of patients in the good treatment response group were with mild or common symptoms, whereas those with poor response had severe or critical symptoms, this data suggested the initial clinical type was associated with the prognosis of COVID‐19. Using the clinical model independently for the treatment response prediction, a good specificity (90.9%) was obtained, whereas a relatively low sensitivity (60.0%) was found when the best cut‐off was 0.847.

In this study, we tracked the dynamic profile of the clinical characteristics of 36 COVID‐19 patients, which further helps understand the potential risk of poor/fair treatment response. The poor/fair group had higher body temperature, lower oxygen saturation, and larger pulmonary lesion percentages compared with the good treatment response group. Moreover, a decreased total lymphocytes, prolonged prothrombin time, increased neutrophil count, D‐dimer, blood urea, and creatinine levels were seen in the poor/fair group, suggesting the more severe cellular immune deficiency, direct effects of the virus, cytokine storm, inflammatory response in this cohort, and more severe myocardia, hepatic, and kidney injuries may indicate poor response to COVID‐19 treatment. These data are consistent with other studies^4^, ^5^ that demonstrate the same alteration trends of these parameters in non‐survivors of COVID‐19.

This study has some limitations. This is a retrospective study with a relatively small sample size. Moreover, the specificity of the model needs to further improve the diagnosis of serve clinical symptoms.

In conclusion, we established a novel, radiomics‐based prognostic model to predict the treatment response to COVID‐19. The nomogram yields a promising predictive value for identifying the potential patients at high risk of fair/poor treatment response, which may assist the decision and management of COVID‐19.

## AUTHOR CONTRIBUTIONS

*Guarantor of integrity of the entire study*: Shisan Bao and Lianping Zhao. *Study concepts and design*: Gang Huang, Zhongyi Hui, Jialiang Ren and Lianping Zhao. *Literature research*: Lianping Zhao, Xing Zhou, Gang Huang and Lijun Chen. *Clinical studies*: Lianping Zhao, Suzhen Lv, Zehao Zhao, Xing Zhou and Lijun Chen. *Experimental studies/data analysis*: Gang Huang, Zhongyi Hui and Shisan Bao. *Statistical analysis*: Ruifang Liu, Yaqiong Cui, Ying Ma and Yalan Han. *Manuscript writing*: All authors. All authors have read and agreed to the published version of the manuscript. So, everyone should be listed as the author.

## CONFLICT OF INTEREST STATEMENT

The authors have no relevant financial or non‐financial interests to disclose.

## ETHICS STATEMENT

The present study was approved by the Human Ethical Committee, Gansu Provincial Hospital (Approval number: 2020‐012).

## INFORMED CONSENT

The requirement for informed consent was waived due to the retrospective nature of the study.

## Supporting information




**Appendix S1.** Radiomics feature extraction methodology and reproducibility
**Appendix S2.** The Statistical Packages of R Software
**Appendix S3.** Radiomics score calculation formula
**Appendix S4.** Methods for calculation of the lung lesion volume and percentage
**Appendix S5.** Definition of Small Area Emphasis feature

**Figure S1.** The detailed pair‐wise relationship between the three statistically significant deferent indicators. CRE = Serum creatinine.

**Figure S2.** The Small Area Emphasis illustrate example.

## Data Availability

The data set supporting the results of this article are included within the article.
