# Supplementary material for: Potential predictive value of CT radiomics features for treatment response in patients with COVID‐19
Source: Clin Respir J. 2023 Mar 21;17(5):394–404. doi: 10.1111/crj.13604 (PMC10214574; doi:10.1111/crj.13604)
Supplement: Supplementary file 1 — Appendix S1. Radiomics feature extraction methodology and reproducibility Appendix S2. The Statistical Packages of R Software Appendix S3. Radiomics score calculation formula Appendix S4. Methods for calculation of the lung lesion volume and percentage Appendix S5. Definition of Small Area Emphasis feature [file CRJ-17-394-s001.docx]

**Appendix 1. Radiomics feature extraction methodology and reproducibility**

In our study, a total of 93 imaging features for each patient were finally extracted from one baseline CT image. All radiomics features were calculated automatically with the PyRadiomics software. The 93 features could be divided into two categories: (a) first-order statistical features (histogram), (b) statistics-based textural features.

The detailed information of these features was available in the documentation for PyRadiomics, <http://PyRadiomics.readthedocs.io/en/latest/>).

*(1) First-order statistics features*

First-order statistics describe the distribution of voxel intensities within the image region defined by the mask through commonly used and basic metrics. A total

of 18 first-order statistics features were extracted.

*(2) statistics-based textural features*

Statistics-based textural features can reflect the homogeneity phenomenon of

the images and the arrangement of the properties that change slowly or periodically on the body surface. Textural features extracted in our study included five types of matrix features, including 24 gray-level co-occurrence matrix (GLCM) features, 16 gray-level run length matrix (GLRLM) features, 16 gray-level size zone matrix (GLSZM) features, 5 Neighboring Gray Tone Difference Matrix (NGTDM), and 14 gray-level dependence matrix (GLDM) features. Determining the texture matrix representations requires the voxel intensity values within the volume of interest (VOI) to be discretized. Voxel intensities were therefore resampled into equally spaced bins using a bin-width of 25 Hounsfield units. This discretization step not only reduces the image noise but also normalizes the intensities across all patients, allowing for a direct comparison of all the calculated textural features between patients.

A GLCM describes the distance and angle of each pixel, which calculates the correlation between two gray levels with certain directions and distances. GLCM can reflect integrated information regarding the direction, interval, amplitude, and frequency of the images. As for GLRLM, the run length metrics quantify the gray level runs in an image. A gray level run is defined as the length in the number of pixels and of the consecutive pixels that have the same gray-level value. A GLSZM describes the amount of homogeneous connected areas within the tumor volume, of a certain size and intensity, thus reflecting the lung heterogeneity at a regional scale.

**Appendix 2. The Statistical Packages of R Software**

The ROC curves were plotted using the “pROC” package. Multivariate logistic regression, nomograms and calibration curves were conducted with the “rms” package. The Hosmer-Lemeshow test was done with the “Resource Selection” package. DCA was performed with the function “dca.R".

All statistical tests were two-sided, and P values of <0.05 were considered significant.

**Appendix 3. Radiomics score calculation formula**

The CT images based Rad-score was calculated for every patient based on the features as follows:

CT Rad-score = -13.4+1.605×glszm_SmallAreaEmphasis -1.42×InitalClinicalType-0.169×CRE

**Appendix 4. Methods for calculation of the lung lesion volume and percentage**

The pulmonary lesion was semi-automatically quantified objectively, using a 3D Slicer software (Version Slicer 4.10.2, <https://www.slicer.org>) and Lung Intelligence Kit software (LK; Version:1.1.0, GE Healthcare, China). Whole lung volume was extracted by Otsu algorithm which was perform on LK software. Lesion volume was extracted by the 3D Slicer software. The procedure was as follows: (1) Load CT images of each patient separately; (2) load lung volume extract by LK software as image mask; (3) Establish a volume file for the lesion, set the threshold for covering all the inflammatory lesion of the lung (the mean threshold range is (-700.93 ± 133.37~ 102.57± 161.89); (4) Apply seed growing and smoothing algorithm in lesion area for removing the noise spot from the remaining lung tissue. Then calculate the percentage of lung lesions in total lung volume by using segment statistics in slicer.

**Appendix 5. Definition of Small Area Emphasis feature**

$$Small Area Emphasis=\frac{\sum_{i=1}^{N_{g}} \sum_{j=1}^{N_{s}} \frac{\boldsymbol{P}\left( i,j \right)}{j^{2}}}{N_{z}}$$

Where $N_{g}$ be the number of discrete intensity values in the image, $N_{s}$ be the number of discrete zone sizes in the image, $N_{z}$ be the number of zones in the ROI and $\boldsymbol{P}\left( i,j \right)$ be the size zone matrix. small area emphasis is a measure of the distribution of small size zones, with a greater value indicative of more smaller size zones and more fine textures.
