# Supplementary figures and images for: Potential predictive value of CT radiomics features for treatment response in patients with COVID‐19
Source: Clin Respir J. 2023 Mar 21;17(5):394–404. doi: 10.1111/crj.13604 (PMC10214574; doi:10.1111/crj.13604)

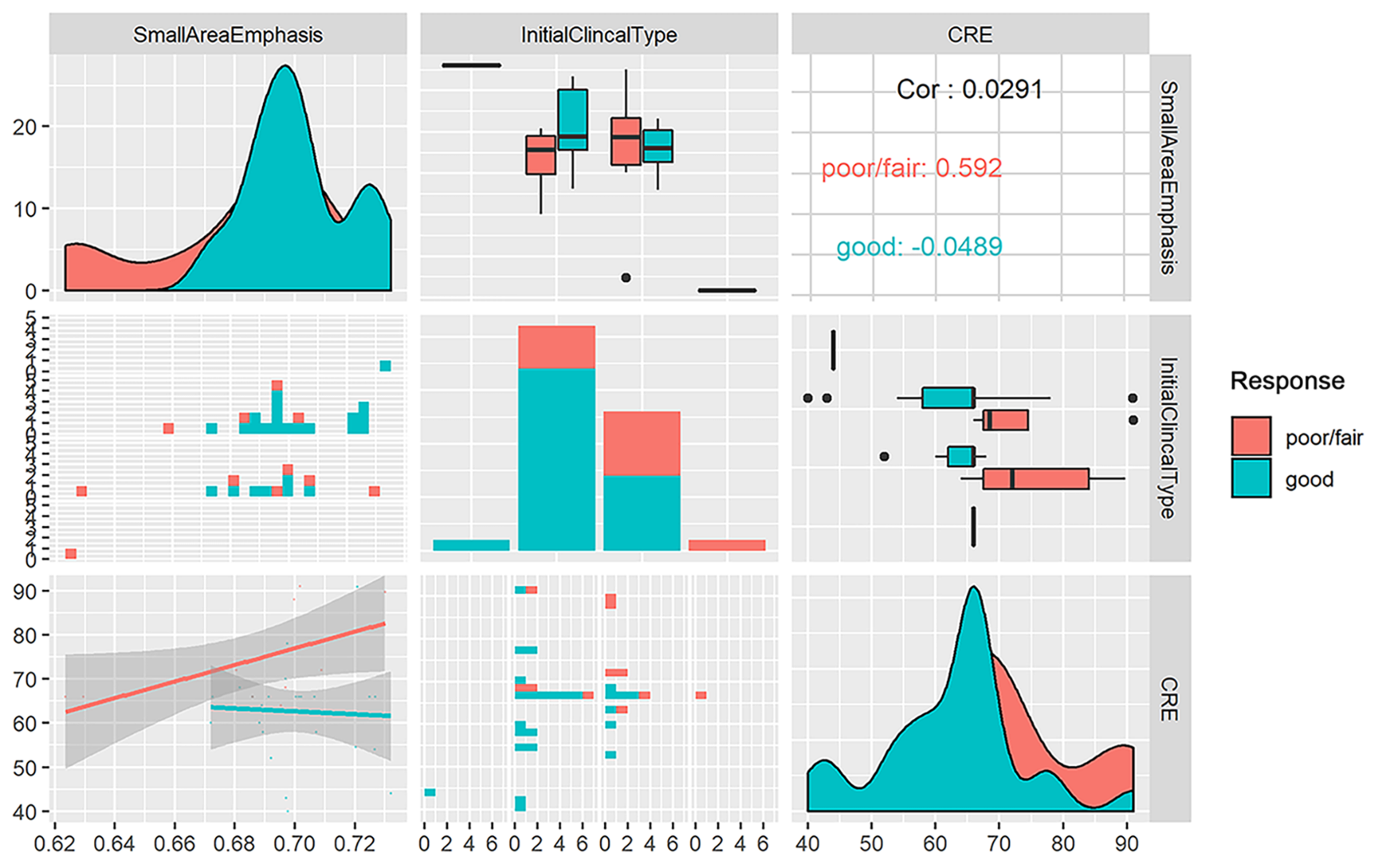

Supplement: Supplementary file 2 — Figure S1. The detailed pair‐wise relationship between the three statistically significant deferent indicators. CRE = Serum creatinine. [file CRJ-17-394-s003.tif]

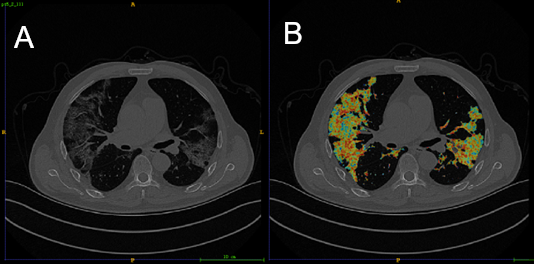

Supplement: Supplementary file 3 — Figure S2. The Small Area Emphasis illustrate example. [file CRJ-17-394-s002.tif]
